# Natural variation in *SlSOS2* promoter hinders salt resistance during tomato domestication

**DOI:** 10.1093/hr/uhac244

**Published:** 2022-10-26

**Authors:** Yechun Hong, Xijin Guan, Xu Wang, Dali Kong, Shuojun Yu, Zhiqiang Wang, Yongdong Yu, Zhen-Fei Chao, Xue Liu, Sanwen Huang, Jian-Kang Zhu, Guangtao Zhu, Zhen Wang

**Affiliations:** Shanghai Center for Plant Stress Biology and Center for Excellence in Molecular Plant Sciences, Chinese Academy of Sciences, Shanghai 200032, China; Institute of Advanced Biotechnology and School of Life Sciences, Southern University of Science and Technology, Shenzhen 518055, China; The AGISCAAS-YNNU Joint Academy of Potato Sciences, Yunnan Normal University, Kunming, China; School of Life Sciences, Anhui Agricultural University, Hefei, Anhui, 230036, China; Shanghai Center for Plant Stress Biology and Center for Excellence in Molecular Plant Sciences, Chinese Academy of Sciences, Shanghai 200032, China; School of Life Sciences, Anhui Agricultural University, Hefei, Anhui, 230036, China; School of Life Sciences, Anhui Agricultural University, Hefei, Anhui, 230036, China; Shanghai Center for Plant Stress Biology and Center for Excellence in Molecular Plant Sciences, Chinese Academy of Sciences, Shanghai 200032, China; Shanghai Center for Plant Stress Biology and Center for Excellence in Molecular Plant Sciences, Chinese Academy of Sciences, Shanghai 200032, China; Shanghai Center for Plant Stress Biology and Center for Excellence in Molecular Plant Sciences, Chinese Academy of Sciences, Shanghai 200032, China; Genome Analysis Laboratory of the Ministry of Agriculture, Agricultural Genomics Institute at Shenzhen, Chinese Academy of Agricultural Sciences, Shenzhen, China; Shanghai Center for Plant Stress Biology and Center for Excellence in Molecular Plant Sciences, Chinese Academy of Sciences, Shanghai 200032, China; Institute of Advanced Biotechnology and School of Life Sciences, Southern University of Science and Technology, Shenzhen 518055, China; The AGISCAAS-YNNU Joint Academy of Potato Sciences, Yunnan Normal University, Kunming, China; School of Life Sciences, Anhui Agricultural University, Hefei, Anhui, 230036, China; Shanghai Center for Plant Stress Biology and Center for Excellence in Molecular Plant Sciences, Chinese Academy of Sciences, Shanghai 200032, China

## Abstract

Increasing soil salinization seriously impairs plant growth and development, resulting in crop loss. The Salt-Overly-Sensitive (SOS) pathway is indispensable to the mitigation of Na ^+^ toxicity in plants under high salinity. However, whether natural variations of *SOS2* contribute to salt tolerance has not been reported. Here a natural variation in the *SlSOS2* promoter region was identified to be associated with root Na^+^/K^+^ ratio and the loss of salt resistance during tomato domestication. This natural variation contains an ABI4-binding *cis*-element and plays an important role in the repression of *SlSOS2* expression. Genetic evidence revealed that *SlSOS2* mutations increase root Na^+^/K^+^ ratio under salt stress conditions and thus attenuate salt resistance in tomato. Together, our findings uncovered a critical but previously unknown natural variation of *SOS2* in salt resistance, which provides valuable natural resources for genetic breeding for salt resistance in cultivated tomatoes and other crops.

## Introduction

Salt stress caused by soil salinization is one of the main threats to global crop yield [1]. Salt stress usually leads to ion toxicity, mainly sodium (Na^+^) toxicity due to its abundance in saline soil. Na^+^ is absorbed by roots and translocated to the shoot tissues, resulting in crop failure. Plants have evolved a series of resistance mechanisms to adapt to high salinity, which comprise restricting Na^+^ uptake, accelerating Na^+^ exclusion, regulating Na^+^ distribution, and adjusting cellular Na^+^ and K^+^ homeostasis [2, 3]. Under salt stress, plants also adjust K^+^ acquisition and distribution to mitigate the toxic effect of Na^+^ by maintaining ion homeostasis [4].

A series of membrane transporters and their regulators are thought to control the balance of Na^+^ and K^+^ and adjust the plant response to salt stress. The Salt-Overly-Sensitive (SOS) pathway is known as a calcium-dependent protein kinase pathway for salt stress signal transduction and Na^+^ tolerance in plants [2]. The Na^+^ stress signal is perceived by the EF-hand calcium-binding protein SOS3 through elevated cytosolic calcium in the root [5]. Subsequently, SOS3 directly interacts with and activates SOS2, a member of the calcineurin B-like interacting protein kinase (CIPK) [6]. The activated SOS2 phosphorylates and activates SOS1, a plasma membrane-localized Na^+^/H^+^ antiporter mediating Na^+^ efflux to diminish cytosolic Na^+^, and thus attenuates Na^+^ toxicity [7]. SOS3 expression is mainly observed in the root, while its paralog SCaBP8/CBL10 is preferentially expressed in the shoot and plays a role parallel to SOS3 [8]. The xylem-localized HKT1 (high-affinity potassium transporter 1) is another important transporter that was first reported to unload Na^+^ from the transpiration stream but also play a role in Na^+^-selective transport in the root and impact Na^+^ distribution in roots and shoots [9–11]. Interestingly, SOS1 is also considered to mediate Na^+^ efflux from root epidermal cells and control Na^+^ transshipment from root to shoot in the parenchyma cells and reduce Na^+^ accumulation in plants, while HKT1 is conducive to unload Na^+^ from the xylem sap and facilitates Na^+^ reflux from shoot to root [3]. Therefore, identification and functional studies of these genes in crops could generate valuable genetic resources for the improvement of salt resistance.

Natural variation in plants reflects the genetic diversity of an identical species during domestication. Mining natural variation has proved to be a powerful approach for revealing the molecular basis of crop mineral composition, a valuable trait in crops [11–14]. Modern commercial tomato varieties are normally less salt-tolerant than wild varieties during artificial selection for large fruits. Our previous study identified a natural variation in an Na^+^/K^+^ transporter gene conferring loss of salt resistance during tomato domestication [15]. In addition, our recent study also revealed natural variations in *SlSOS1* conferring the loss of salt resistance in cultivated tomato during domestication [16]. Importantly, natural variations in *ZmSOS1* and *ZmSOS3* genes have been identified and applied to improve the salt-resistance trait in maize [17]. However, natural variations in *SOS2* for salt resistance in plants have not been reported. Herein, we identified a natural variation in the *SlSOS2* promoter region associated with the root Na^+^/K^+^ ratio and the loss of salt resistance during domestication. An ABI4-binding *cis*-element was found to be located in this significant variation and inclusion of this element reduces *SlSOS2* expression. We further revealed that knockout mutations of *SlSOS2* in tomato result in a phenotype of hypersensitivity to high salinity. Consistently, loss of *SlSOS2* function resulted in an increased level of Na^+^ but a decreased level of K^+^, and thus an increased Na^+^/K^+^ ratio in roots under salt stress conditions.

## Results

### A natural variation in *SlSOS2* promoter is associated with root Na^+^/K^+^ ratio and tomato domestication in response to salt stress

**Figure 1:**
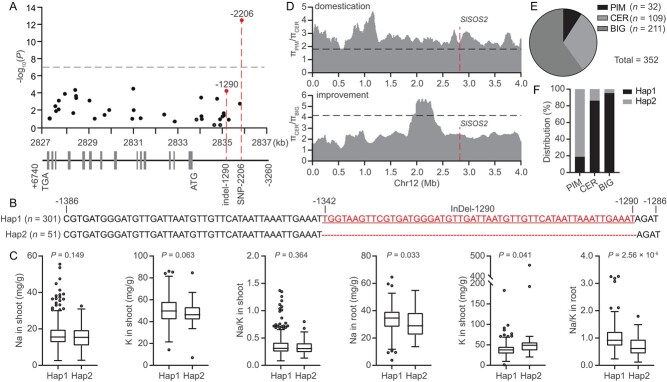
Identification of natural variations in *SlSOS2* associated with root Na^+^/K^+^ ratio under salt stress. (A) Association mapping of the *SlSOS2* variants and root Na^+^/K^+^ ratio. Red dashed lines indicate significant variations. The gray dashed line shows the Bonferroni-adjusted significance threshold (*P* = 1.0 × 10^−7^). (B) Two haplotypes of *SlSOS2* in the tomato population according to indel-1290. The sequences show the indel-1290 of the Hap1 and Hap2 promoters. The red nucleotides and dots represent nucleotide variations. (C) Distributions of Na^+^ and K^+^ contents and ratio are displayed as box plots in two haplotype groups. The box indicates the range of the data percentiles using the Tukey method, the middle line indicates the median, the outer dots refer to outlying values, and the whiskers are the interquartile range. *P*-values were determined by Student’s *t-*test. (D) Distribution of nucleotide diversity in PIM, CER, and BIG varieties on chromosome 12. The position of *SlSOS2* indicated by the red dashed line is within a domestication and improvement sweep. π, nucleotide diversity. (E) Distribution of 352 accessions, including 32 PIM, 109 CER, and 211 BIG. (F) Frequencies of two *SlSOS2* alleles in three groups.

Tomato, as one of the global leading vegetable crops, possesses an assembled genome with publicly available sequences [18–20]. Intriguingly, modern cultivated tomato accessions gradually lost the salt resistance of their original wild varieties during artificial selection for large fruits [15]. Considering the important and conserved roles of the canonical SOS pathway in plant response to salt stress [21], whether natural variations in the *SOS2* gene contribute to this process was investigated. Based on the phylogenetic tree of CIPK proteins, we found that SlCIPK20/SlSOS2, AtCIPK24/AtSOS2, and ZmCIPK24a/ZmSOS2 clustered in the same clade (Supplementary Data Fig. 1 and Supplementary Data Table 1), suggesting that SOS2 is evolutionarily conserved in plants. Moreover, SlSOS2 was reported to interact with SlSOS1 to facilitate Na^+^ transport in yeast cells, and overexpression of *SlSOS2* in plants showed a salt-resistance phenotype [22]. Hence, we collected 364 tomato accessions from the relateds population of origin, comprising 35 wild accessions of *Solanum pimpinellifolium* (PIM), 115 domesticated accessions of *Solanum lycopersicum* var. *cerasiforme* (CER), and 214 improved accessions of *Solanum lycopersicum* (BIG) (Supplementary Dataset 1). All the sequence variations, including SNPs (single nucleotide polymorphisms) and indels (insertions and deletions), in the promoter and genomic regions of *SlSOS2* among the 352 accessions were then identified according to the reference genome [18]. Association analysis showed that only one variation (SNP-2206) in the *SlSOS2* promoter region was strikingly associated with root Na^+^/K^+^ ratio under salt stress (*P* = 3.32 × 10^−13^), but this variable region does not contain motifs potentially impacting the expression of *SlSOS2* (Fig. 1A and Supplementary Dataset 2), and thus was not selected for further study. According to the resequencing result of the *SlSOS2* promoter region, a 53-bp indel (named indel-1290) upstream of the ATG start codon was identified by comparing cultivated accessions (representative BIG, TS-577) and wild accessions (representative PIM, TS-21) (Supplementary Dataset 1). The variant promoter name *SlSOS2^TS-577^pro* designates promoters with insertions and *SlSOS2^TS-21^pro* designates promoters with deletions. We then classified the 364 accessions into three haplotypes based on indel-1290 in the *SlSOS2* promoter. The cultivated *SlSOS2^TS-577^* promoter belongs to Hap (haplotype) 1 (*n* = 301) with insertions, while the wild *SlSOS2^TS-21^* promoter is representative of Hap2 (*n* = 51) without insertions (Fig. 1B). The accessions containing a heterozygous *SlSOS2* promoter were classified as the third group, Hap3 (*n* = 12) (Supplementary Dataset 1). We statistically analyzed the contents of Na^+^ and K^+^ in the 364 accessions and found that the accessions in Hap1 showed a higher Na^+^ level and a lower K^+^ level in roots but not shoots compared with Hap2, thus increasing the root Na^+^/K^+^ ratio of Hap1. However, the accumulation of Na^+^ and K^+^ in Hap1 was not significantly different from that of Hap2 in shoots (Fig. 1C and Supplementary Dataset 1). These results indicated that indel-1290 is also strongly associated with root Na^+^/K^+^ ratio under salt stress. Because the root Na^+^/K^+^ ratio is inversely correlated with salt resistance in tomato [15], Hap1 and Hap2 were designated as the sensitive and tolerant alleles of *SlSOS2*, respectively. In addition, the resided region of this locus was discovered to be associated with domestication rather than an improvement sweep (Fig. 1D). Consistently, the distribution of these two alleles in the PIM, CER, and BIG groups of the 352 accessions further indicated that the frequency of the tolerant allele gradually decreased from PIM to CER and then to BIG (Fig. 1E and F). These results indicated that this natural variation in the *SlSOS2* promoter region is clearly associated with the Na^+^/K^+^ ratio in the root, which possibly contributes to the loss of salt resistance during tomato domestication.

### Transcription factor ABI4 directly binds to *SlSOS2* promoter to regulate its expression under salt stress

**Figure 2:**
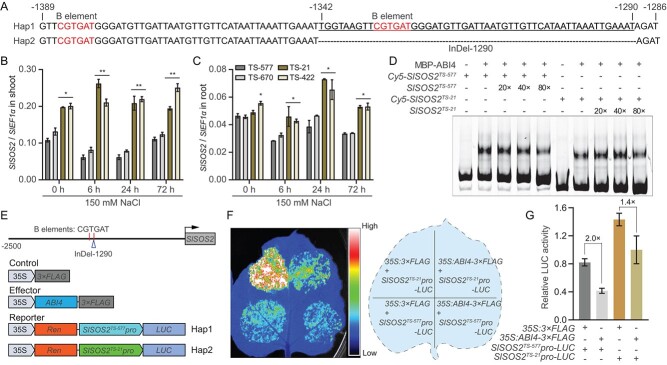
Variation in the *SlSOS2* promoter contributes to its expression under salt stress. (A) The sequence of B elements was identified in the Hap1 and Hap2 promoters. The nucleotides of B elements are highlighted in red. (B, C) Transcript levels of *SlSOS2* were determined by qRT–PCR analysis in shoots (B) and roots (C) of TS-577, TS-670, TS-21, and TS-422 after treatment with 150 mM NaCl for the indicated number of hours. Error bars indicate ± standard deviation (*n* = 3). ^*^P < .05 and ^**^P < .01, Student’s *t*-test. (D) Binding capacity of ABI4 to the B elements in the *SlSOS2* promoter. Reciprocal competitive EMSA to examine the binding of MBP–ABI4 fusion protein to the fragments of the promoter region containing the B elements of *SlSOS2^TS-577^* and *SlSOS2^TS-21^* was executed using Cy5-labeled probes and unlabeled competitors. (E) Schematic diagrams of the effector and reporter constructs were used in the transactivation assay. (F) Transactivation assay of *SlSOS2* promoters in tobacco leaves. (G) Relative LUC activity indicated that ABI4 represses the expression of *SlSOS2*. Values represent ± standard deviation (*n* = 6).

Promoter analysis further uncovered that indel-1290 is within a known B element with the core sequence of CGTGAT (Fig. 2A), and the promoters containing this *cis*-element were found to be recognized and depressed by the ABI4 (ABA INSENSITIVE 4) transcription factors in response to stress conditions [23–25]. We thus chose the representative promoters of *SlSOS2^TS-577^* and *SlSOS2^TS-21^* from the two haplotypes for further study. The *SlSOS2^TS-577^pro* with the 53-bp insertion of indel-1290 possessed two B elements, whereas the deletion of indel-1290 in *SlSOS2^TS-21^pro* resulted in only one B element in the promoter region (Fig. 2A). To test whether this variation contributes to *SlSOS2* expression, we examined the transcript levels of *SlSOS2* in response to salt stress treatment. The results revealed that the expression of *SlSOS2* was enhanced in the Hap2 varieties by salt stress, whereas this upregulation was not observed in the Hap1 accessions (Fig 2B and C). The binding capacity of the B elements with ABI4 was determined by reciprocal competitive electrophoretic mobility shift assay (EMSA). The results showed specific binding of ABI4 to the fragments containing B elements in both *SlSOS2^TS-577^pro* and *SlSOS2^TS-21^pro*, suggesting that the presence of one or two B elements in the *SlSOS2* promoter region has no effect on ABI4 binding efficiency (Fig. 2D). Therefore, we speculated that the lower expression of *SlSOS2* in Hap1 than in Hap2 was caused by reduced transactivation. To assess this assumption, we analyzed the transcriptional activity of *SlSOS2pro* with ABI4, which is known as a salt-inducible transcription factor in plants suppressing the expression of target genes [25, 26]. The transcriptional activity assay showed that both *SlSOS2^TS-577^pro* and *SlSOS2^TS-21^pro* fused to the *LUC* (*Luciferase*) gene were significantly depressed by the expression of effector gene *ABI4*, but the transcriptional activity of *SlSOS2^TS-577^pro* was lower than that of *SlSOS2^TS-21^pro*, indicating that ABI4 is capable of functioning as a transcriptional repressor of *SlSOS*2 in tomato (Fig 2E–G). This observation is consistent with the transcription levels of *SlSOS2* in the two haplotypes under normal and salt-stress conditions, supporting the idea that the variation in the *SlSOS2* promoter is conducive to controlling *SlSOS2* transcription in tomato.

### Expression of *SlSOS2* improves salt resistance in plants

**Figure 3:**
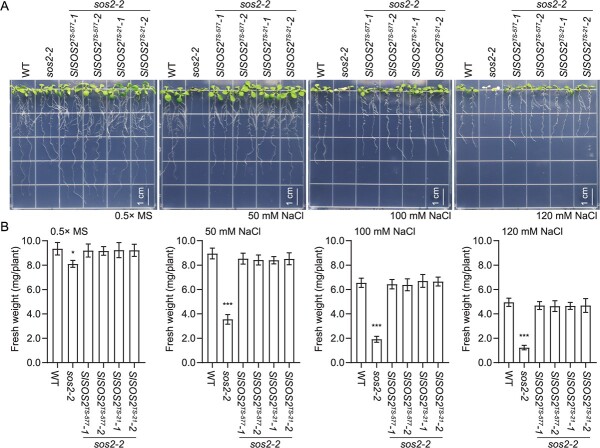
Functional complementation of *sos2-2* mutant using the two *SlSOS2* alleles under salt stress. (A) Salt tolerance of Col-0, *sos2-2*, and transgenic plants. Seven-day-old seedlings were transplanted to 0.5× MS medium with 0, 50, 100, or 120 mM NaCl for an additional 7 days. Scale bars = 1 cm. (B) Determination of fresh weight of seedlings described in (A). Error bars are means ± standard deviation (*n* = 20). ^*^P < .05 and ^***^P < .001, Student’s *t*-test. *SlSOS2^TS-577^-1* and *SlSOS2^TS-577^-2*, two independent transgenic lines of *35S:SlSOS2^TS-577^-YFP* in *sos2-2* mutant; *SlSOS2^TS-21^-1* and *SlSOS2^TS-21^-2*, two independent transgenic lines *35S:SlSOS2^TS-577^-YFP* in *sos2-2* mutant.

Considering that variations in the *SlSOS2* gene coding region may also contribute to various salt resistances in plants, we introduced *SlSOS2* genes of two haplotypes into *Arabidopsis sos2-2* mutant plants to test whether these two *SlSOS2* alleles could recover the salt-sensitive phenotype of the *sos2-2* mutant. Two independent transgenic lines of *35S:SlSOS2^TS-577^-YFP* were designated *SlSOS2^TS-577^-1* and *SlSOS2^TS-577^-2*, while two independent transgenic lines of *35S:SlSOS2^TS-21^-YFP* were named as *SlSOS2^TS-21^-1* and *SlSOS2^TS-21^-2* (Supplementary Data Fig. 2). A physiological assay revealed that both *SlSOS2^TS-577^* overexpression and *SlSOS2^TS-21^* overexpression are capable of complementing the salt-hypersensitive phenotype of *sos2-2* mutant plants (Fig. 3), indicating that the variations in the *SlSOS2* coding region are not responsible for the difference in salt resistance in plants. These results also suggest that, like *Arabidopsis SOS2* [27], *SlSOS2* plays a pivotal role in salt resistance.

**Figure 4:**
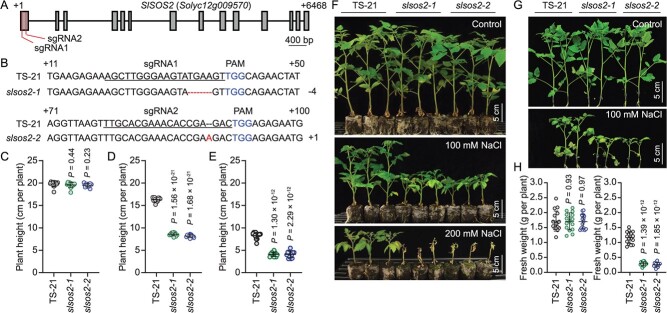
Knockout of *SlSOS2* obviously decreases salt resistance in tomato*.* (A) Mutants of *SlSOS2^TS-21^* were generated by the CRISPR/Cas9 system with two independent single-guide RNAs (sgRNA1 and sgRNA2). (B) Nucleotides of *SlSOS2* in wild type (TS-21) and *slsos2-1* and *slsos2-2* mutants are shown. The underlines pinpoint sgRNA-targeted sequences. The blue nucleotides indicate the protospacer-adjacent motif (PAM). The deletion is indicated by a dashed line and the insertion is labeled in red. (C–E) Measurement of plant height for TS-21, *slsos2-1*, and *slsos2-2* described in (F). Error bars are means ± standard deviation (*n* = 12 plants of each genotype). (F) Salt resistance of TS-21 wild-type and *slsos2* mutants. Eighteen-day-old TS-21, *slsos2-1*, and *slsos2-2* plants were treated with 100 or 200 mM NaCl in soil for 3 weeks. Control tomato plants were grown under normal conditions for 4 weeks. Scale bars = 5 cm. (G) Eighteen-day-old TS-21, *slsos2-1*, and *slsos2-2* plants grown in 0.25× MS liquid medium with 100 mM NaCl for 7 days. (H) Determination of fresh weight of the TS-21, *slsos2-1*, and *slsos2-2* plants shown in (G). Scale bars = 5 cm. Error bars are means ± standard deviation (*n* = 16 plants of each genotype). *P*-values were determined by Student’s *t*-test.

We further generated *SlSOS2* mutations in salt-tolerant accession TS-21 using the CRISPR/Cas9 system and two mutant alleles were designated *slsos2-1* and *slsos2-2* (Fig. 4A and B). Phenotype analysis showed that *slsos2-1* and *slsos2-2* mutants displayed clearly reduced salt resistance when compared with the wild-type TS-21 in soil (Fig. 4C–F). In addition, we validated the phenotype of *slsos2* mutants in liquid culture, and both mutant alleles also were more sensitive to salt stress than wild-type plants (Fig. 4G and H). These results strongly support the role of *SlSOS2* in salt tolerance in tomato.

### Genetic mutations in *SlSOS2* reduce salt resistance caused by increasing root Na^+^/K^+^ ratio in tomato

**Figure 5:**
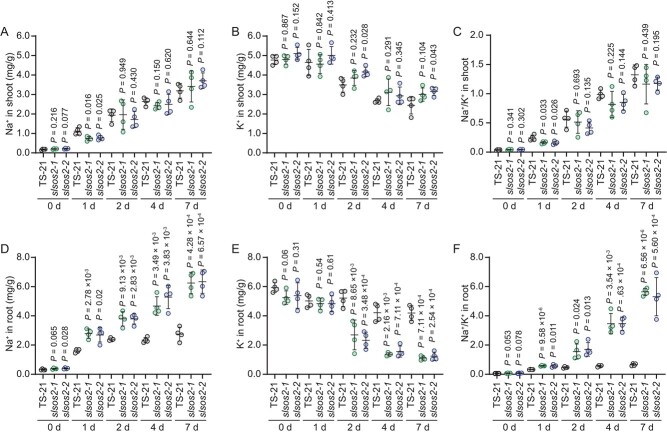
SlSOS2 controls Na^+^ and K^+^ accumulation in tomato during salt stress. (A–C) Na^+^ (A) and K^+^ (B) contents and Na^+^/K^+^ ratio (C) in shoots of 28-day-old TS-21, *slsos2-1*, and *slsos2-2* plants treated with 100 mM NaCl for the indicated number of days. Data are shown as means ± standard deviation (*n* = 4 biological repeats). (D–F) Contents of Na^+^ (D) and K^+^ (E) and Na^+^/K^+^ ratio (F) and in roots of 28-day-old *slsos2-1*, *slsos2-2*, and TS-21 wild-type plants treated with 100 mM NaCl for the indicated number of days. Error bars are means ± standard deviation (*n* = 4 biological repeats). *P*-values were determined by Student’s *t*-test.

As a serine/threonine protein kinase, SOS2 phosphorylates and activates Na^+^/H^+^ antiporter SOS1 to adjust Na^+^ accumulation in *Arabidopsis* [28, 29]. Hence the reduction of salt resistance in *slsos2* mutants might be associated with Na^+^ accumulation in roots. To test this hypothesis, we determined the contents of Na^+^ and K^+^ in shoots and roots of TS-21 and *slsos2* mutant plants exposed to salt stress. Ion content analysis indicated that *slsos2-1* and *slsos2-2* mutants displayed a similar level of Na^+^ and K^+^ in shoots when compared with wild-type plants before and after salt stress treatment, except for 1-day salt treatment, with which the mutants accumulated apparently lower Na^+^ in shoots than the wild-type (Fig. 5A and B). Consequently, the Na^+^/K^+^ ratio in shoots of wild-type and mutants showed no significant difference except for the 1-day salt treatment, with which the mutant showed lower Na^+^/K^+^ ratio than wild-type in shoots (Fig. 5C). Interestingly, the accumulation of Na^+^ in roots was strikingly higher in the mutants than in wild-type TS-21 under salt stress (Fig. 5D). Greater reduction of K^+^ content in the mutants than in wild-type was clearly observed in the roots after salt treatment for ≥2 days (Fig. 5E). Thus, the mutant roots exhibited a markedly higher Na^+^/K^+^ ratio than the roots of wild-type TS-21 (Fig. 5F). These results suggested that SlSOS2 governs Na^+^ and K^+^ redistribution in tomato roots and shoots to improve salt resistance. Collectively, our findings revealed that the natural variation in the promoter of *SlSOS2* enhances its transcriptional repression by ABI4, leading to reduced *SlSOS2* expression and decreased salt resistance in the cultivated tomato during domestication with selection for large fruits. The variation in the wild *SlSOS2* allele provides valuable natural resources for improvement of salt resistance in tomato.

## Discussion

Soil salinization resulting in the loss of crop production is one of the major threats to agriculture worldwide [30]. Plants have evolved mechanisms to adapt to high salinity, which include the regulation of ion homeostasis to mitigate ion toxicity [29]. The SOS signaling pathway was identified as a crucial pathway to maintain ion homeostasis and thus reduce Na^+^ toxicity [2, 28]. Genetic and molecular mechanisms acquired from *Arabidopsis* are potentially applicable in crops for improvement of stress resistance. On the other hand, mining and mechanistic studies of the natural genetic variations in crops could provide valuable resources for breeding for stress tolerance [31–33]. Our previous study identified two natural variations in the *SlSOS1* promoter associated with the root Na^+^/ K^+^ ratio and salt resistance in tomato [16]. Soon after this report, the natural variations of *ZmSOS1* and *ZmSOS3* were found to contribute to shoot Na^+^ contents and salt resistance in maize [17]. However, natural genetic variations in *SOS2* and their contributions to salt resistance in plants remain largely unknown.

To this end, we analyzed the resequencing data of tomato salt-resistant and sensitive accessions and found that a natural variation in the *SlSOS2* promoter region with a 53-bp deletion contributes to the salt-resistance phenotype of wild tomato varieties (Fig. 1B). According to this variation, three haplotypes were classified as Hap1–3; Hap3 (including 12 accessions) was not considered in this study because it contained a heterozygous *SlSOS2* promoter (Supplementary Dataset 1). The Hap1 group showed a higher level of Na^+^ but less K^+^ in roots when compared with the Hap2 group; thus there was a significantly higher root Na^+^/K^+^ ratio in the Hap1 plants than in the Hap2 plants under salt stress (Fig. 1C). These findings suggest that the 53-bp indel as a natural variation is clearly associated with root Na^+^/K^+^ ratio in tomato. Furthermore, promoter analysis revealed that the 53-bp insertion of Hap1 contains a B element. As a result, *SlSOS2^TS-577^pro* possesses two B elements, while *SlSOS2^TS-21^* only has one B element in the promoter region (Fig. 2A). Because salt-inducible ABI4 can bind to B elements and depress the expression of target genes [25, 26], we tested the binding affinity and transcriptional activity of two *SlSOS2* alleles with the ABI4 transcription factor. The results indicated that the promoters of the two *SlSOS2* alleles were able to be recognized by ABI4, whereas the transcriptional repression of *SlSOS2^TS-577^* by ABI4 was significantly stronger than that of *SlSOS2^TS-21^* (Fig. 2C–G). These results are consistent with the expression pattern of two *SlSOS2* alleles under normal and salt-stress conditions (Fig. 2B and C), supporting the idea that the variation in *SlSOS2* promoter is conducive to enhanced expression in wild tomato.

In the SOS pathway, the Na^+^/H^+^ antiporter SOS1 plays a role in reducing Na^+^ toxicity through modulating the Na^+^ distribution between shoots and roots via xylem loading [34]. SOS2 is essential to phosphorylate and activate SOS1, which is inactive in a resting state by autoinhibition [35, 36]. Our data showed a markedly higher root Na^+^/K^+^ ratio in *slsos2* mutants than in the wild-type TS-21 under salt stress, which resulted from more Na^+^ but less K^+^ accumulation in roots of the mutants compared with wild-type plants (Fig. 5). These results suggested that SlSOS2 is also involved in Na^+^ and K^+^ homeostasis in tomato roots under salt stress conditions. Moreover, a physiological assay revealed that the *slsos2* mutants were distinctly more sensitive to high salinity than wild-type plants (Fig. 4), further supporting the idea that *SlSOS2* plays a vital role in salt resistance in tomato. Collectively, our findings indicate that the natural variation in the promoter of *SlSOS2* gaining an additional ABI4-binding *cis*-element contributes to increased transcriptional inhibition of *SlSOS2* expression and decreased salt resistance in the cultivated tomato in exchange for selection for large fruits during domestication. The variation in the wild *SlSOS2* promoter provides a valuable natural genetic resource for improvement of salt resistance in tomato.

## Materials and methods

### Plant materials and growth conditions

The tomato population used in this study was included in our previous studies [15]. Tomato seeds were germinated in 0.25× MS (Murashige and Skoog) medium with 0.6% (w/v) agar at 23°C after sterilization. Seven-day-old seedlings were transplanted to 0.25× MS liquid medium (pH 5.9) or soil and grown in a growth room at 23°C with a 16-hours light/8-hours dark period. To assay salt resistance, 18-day-old tomato plants were treated with different concentrations of NaCl for the indicated number of days, and the plants were then subjected to determination of plant height and fresh weight.

### Electrophoretic mobility shift assay

The full-length coding sequence of the *Arabidopsis ABI4* gene was amplified and cloned into the pMAL-c5x vector. This construct was then transformed into *Escherichia coli* to express MBP–ABI4 fusion protein. The fusion protein was purified as described previously [37]. The B elements were identified by analyzing the *SlSOS2* promoter using the PlantCARE database [38]. The EMSA was carried out as previously described [16]. In brief, the binding affinity of ABI4 to the B elements in the promoters of the *SlSOS2^TS-577^* and *SlSOS2^TS-21^* genes was determined using Cy5-labeled DNA fragments containing B elements mixed with 1.5 mg of recombinant protein in binding buffer. Mixtures replenished with unlabeled DNA fragments were employed to test competitive binding. The Cy5 signals were examined with a Starion FLA-9000 (FujiFilm, Japan) after electrophoresis with native polyacrylamide gels.

### Transactivation assay

Promoter fragments of ~2500 bp upstream of the start codon of *SlSOS2^TS-21^* or *SlSOS2^TS-577^* were cloned into the pGreenII-0800-LUC vector. The full-length coding sequence from *Arabidopsis* ABI4 was cloned into pCAMBIA1305 to generate the *ABI4-3 × FLAG* construct. The constructs were subsequently introduced into *Agrobacterium* stain GV3101. *Agrobacterium* cells containing different constructs were combined and infiltrated into *Nicotiana benthamiana* leaves through the method described previously [39]. After culturing for 48 hours, the leaves were injected with 1 μM luciferin and kept in darkness for 5–20 minutes in a growth room. The luminescence signals were examined with a cooled CCD imaging system (Tanon 5200).

### Generation of tomato mutants

The CRISPR/Cas9 system was constructed as described previously [40]. A 19-bp length of single-guide RNA (sgRNA) upstream of the PAM (protospacer adjacent motif, NGG) fragment was cloned into pCAMBIA1300 vector harboring a Cas9 expression cassette. To target the *SlSOS2* gene, the sgRNA was governed by the *AtU6* promoter. Two constructs were then introduced into a wild accession, TS-21, to generate *slsos2* mutant plants. The homozygous *slsos2* alleles were identified from the T0 or T1 generation via PCR-based Sanger sequencing.

### Determination of Na^+^ and K^+^ contents

Determination of contents of Na^+^ and K^+^ in shoots and roots of the tomato population was as described previously [14]. To measure the contents of Na^+^ and K^+^ in *slsos2* mutants and wild-type (TS-21), 18-day-old plants grown in 0.25× MS liquid medium were supplemented with 100 mM NaCl for the indicated number of days. Shoot and root tissues were collected and then rinsed four times with 20 μM EDTA to remove any contaminants before drying at 65°C for 2 days. Subsequently, dry samples were digested by 1 mL of nitric acid at 115°C for 3 hours and diluted to 10 mL using deionized water. Indium was used as the internal standard. The contents of Na^+^ and K^+^ of diluted samples were determined by inductively coupled plasma mass spectrometry (NexION 350D; PerkinElmer) coupled to an Apex desolvation system and an SC-4 DX autosampler (Elemental Scientific, Omaha, NE, USA).

### Quantitative real-time PCR

mRNA in plant tissues was extracted with a Total RNA Extraction Kit (Hua Yue Yang) and reverse-transcribed with a HiScript II 1st Strand cDNA Synthesis Kit (Vazyme). Quantitative real-time PCR was carried out using AceQ qPCR SYBR Green Master Mix (Vazyme). Primers used in this study are shown in Supplementary Data Table 2.

## Acknowledgements

This work was supported by the National Natural Science Foundation of China (grant 32000206 to Z.W.) and the Natural Science Foundation of Anhui Province (grant 2208085Y08 to Z.W.).

## Author contributions

Z.W., G.Z., S.H., and J.-K.Z. conceived the project; Z.W. and G.Z. designed the experiments and analyzed the data; Z.W. and Y.H. performed most of the experiments; G.Z. and X.G. carried out the association mapping; X.W., Z.Q.W., D.K., S.Y., Y.Y., Z.-F.C., and X.L. provided technical assistance; Z.W. and Y.H. drafted the article; Z.W. and G.Z. revised and finalized the manuscript.

## Data availability

The authors declare that all the data supporting the findings of this study are available within the paper and its supplementary information files.

## Conflict of interest

The authors declare no competing financial interests.

## Supplementary data

Supplementary data is available at *Horticulture Research* online.

## Supplementary Material

Web_Material_uhac244
