# Supplementary material for: Natural variation in SlSOS2 promoter hinders salt resistance during tomato domestication
Source: Hortic Res. 2022 Oct 26;10(1):uhac244. doi: 10.1093/hr/uhac244 (PMC9832868; doi:10.1093/hr/uhac244)
Supplement: Web_Material_uhac244 [file web_material_uhac244.zip › Supplemental Figure Legend-R1.docx]

**Supplemental Figure 1.** Phylogenetic tree of CIPK proteins in plants.

(A) Phylogenetic analysis was performed using the amino acid sequences of CIPK proteins from tomato, *Arabidopsis* and maize. The sequences were clustered using ClustalW and the unrooted tree was established using the neighbor-joining method in Mega. The SlSOS2 and its homologous proteins were outlined in red. The gene ID of *CIPKs* used in the phylogenetic analysis were shown in the Supplemental Table 1.

**Supplemental Figure 2.** Characterization of transgenic plants expressing *SlSOS2* in *sos2-2* mutant plants.

(A) The transcript levels of *SlSOS2* in Col-0, *sos2-2*, and four transgenic lines were determined by qRT-PCR. Error bars are means ± SD (n = 3). *SlSOS2^TS-577^-1* and *SlSOS1^TS-577^-2*, two independent transgenic lines of *35S:SlSOS2^TS-577^-YFP* in *sos2-2* mutant; *SlSOS2^TS-21^-1* and *SlSOS1^TS-21^-2*, two independent transgenic lines *35S:SlSOS2^TS-577^-YFP* in *sos2-2* mutant. *** *P* < 0.001, Student’s t-tests.
