# Supplementary material for: Natural variation in SlSOS2 promoter hinders salt resistance during tomato domestication
Source: Hortic Res. 2022 Oct 26;10(1):uhac244. doi: 10.1093/hr/uhac244 (PMC9832868; doi:10.1093/hr/uhac244)
Supplement: Web_Material_uhac244 [file web_material_uhac244.zip › Supplemental Table 1-R1.docx]

**Supplemental Table 1. List of *CIPK* genes in tomato and their homologous genes in *Arabidopsis* and maize.**

| **Name** | **Gene ID** | **Name** | **Gene ID** | **Name** | **Gene ID** |
| --- | --- | --- | --- | --- | --- |
| *SlCIPK1* | solyc01g008850 | *AtCIPK3* | At2g26980 | *ZmCIPK33* | Zm00001d052416 |
|  |  |  |  | *ZmCIPK3* | Zm00001d028949 |
|  |  |  |  | *ZmCIPK44* | Zm00001d022547 |
| *SlCIPK2* | solyc02g021440 | *AtCIPK23* | At1g30270 | *ZmCIPK23* | Zm00001d018799 |
| *SlCIPK3* | solyc02g072530 | *AtCIPK12* | At4g18700 | *ZmCIPK12* | Zm00001d038690 |
|  |  |  |  | *ZmCIPK18* | Zm00001d043389 |
|  |  |  |  | *ZmCIPK19* | Zm00001d010743 |
|  |  |  |  | *ZmCIPK37* | Zm00001d012696 |
| *SlCIPK4* | solyc02g072540 | *AtCIPK20* | At5g45820 | *ZmCIPK5* | Zm00001d008901 |
| *SlCIPK5* | solyc03g005330 | *AtCIPK7* | At3g23000 | *ZmCIPK4* | Zm00001d033316 |
|  |  |  |  | *ZmCIPK7* | Zm00001d041422 |
|  |  |  |  | *ZmCIPK30* | Zm00001d012697 |
| *SlCIPK6* | solyc03g006110 | *AtCIPK20* | At5g45820 | *ZmCIPK5* | Zm00001d008901 |
| *SlCIPK7* | solyc04g076810 | *AtCIPK8* | At4g24400 | *ZmCIPK8* | Zm00001d044553 |
|  |  |  |  | *ZmCIPK31* | Zm00001d000080 |
| *SlCIPK8* | solyc05g047600 | *AtCIPK9* | At1g01140 | *ZmCIPK9* | Zm00001d027455 |
|  |  |  |  | *ZmCIPK32* | Zm00001d048460 |
| *SlCIPK9* | solyc05g052270 | *AtCIPK10* | At5g58380 | *ZmCIPK22* | Zm00001d038048 |
| *SlCIPK10* | solyc05g053210 | *AtCIPK1* | At3g17510 | *ZmCIPK27* | Zm00001d050422 |
|  |  |  |  | *ZmCIPK28* | Zm00001d020497 |
|  |  |  |  | *ZmCIPK21* | Zm00001d006944 |
|  |  |  |  | *ZmCIPK34* | Zm00001d022272 |
| *SlCIPK11* | solyc05g007430 | *AtCIPK10* | At5g58380 | *ZmCIPK11* | Zm00001d043038 |
| *SlCIPK12* | solyc06g007440 | *AtCIPK11* | At2g26980 | *ZmCIPK13* | Zm00001d008899 |
|  |  |  |  | *ZmCIPK29* | Zm00001d022451 |
|  |  |  |  | *ZmCIPK36* | Zm00001d007166 |
| *SlCIPK13* | solyc06g068450 | *AtCIPK25* | At5g25110 | *ZmCIPK16* | Zm00001d005748 |
|  |  |  |  | *ZmCIPK20* | Zm00001d020496 |
| *SlCIPK14* | solyc06g082440 | *AtCIPK25* | At5g25110 | *ZmCIPK13* | Zm00001d008899 |
|  |  |  |  | *ZmCIPK29* | Zm00001d022451 |
|  |  |  |  | *ZmCIPK36* | Zm00001d007166 |
| *SlCIPK15* | solyc07g005440 | *AtCIPK6* | At4g30960 | *ZmCIPK6* | Zm00001d032155 |
|  |  |  |  | *ZmCIPK27* | Zm00001d050422 |
|  |  |  |  | *ZmCIPK28* | Zm00001d020497 |
| *SlCIPK16* | solyc08g067310 | *AtCIPK5* | At5g10930 | *ZmCIPK16* | Zm00001d005748 |
|  |  |  |  | *ZmCIPK20* | Zm00001d020496 |
| *SlCIPK17* | solyc09g018280 | *AtCIPK14* | At5g01820 | *ZmCIPK13* | Zm00001d008899 |
|  |  |  |  | *ZmCIPK29* | Zm00001d022451 |
|  |  |  |  | *ZmCIPK36* | Zm00001d007166 |
| *SlCIPK18* | solyc09g083100 | *AtCIPK7* | At3g23000 | *ZmCIPK4* | Zm00001d033316 |
|  |  |  |  | *ZmCIPK7* | Zm00001d041422 |
|  |  |  |  | *ZmCIPK30* | Zm00001d012697 |
| *SlCIPK19* | solyc11g062410 | *AtCIPK3* | At2g26980 | *ZmCIPK33* | Zm00001d052416 |
|  |  |  |  | *ZmCIPK3* | Zm00001d028949 |
|  |  |  |  | *ZmCIPK44* | Zm00001d022547 |
| *SlCIPK20* | solyc12g009570 | *AtCIPK24* | At5g35410 | *ZmCIPK24a* | Zm00001d036879 |
|  |  |  |  | *ZmCIPK24b* | Zm00001d000407 |
| *SlCIPK21* | solyc12g010130 | *AtCIPK6* | At4g30960 | *ZmCIPK6* | Zm00001d032155 |
|  |  |  |  | *ZmCIPK27* | Zm00001d050422 |
|  |  |  |  | *ZmCIPK28* | Zm00001d020497 |
| *SlCIPK22* | solyc12g098910 | *AtCIPK1* | At3g17510 | *ZmCIPK1* | Zm00001d040567 |
|  |  |  |  | *ZmCIPK17* | Zm00001d024762 |
|  |  |  |  | *ZmCIPK21* | Zm00001d006944 |
|  |  |  |  | *ZmCIPK34* | Zm00001d022272 |

Note: *SlCIPK*, *CIPK* genes in tomato; *AtCIPK*, *CIPK* genes from *Arabidopsis thaliana*; *ZmCIPK*, *CIPK* genes of maize.
