# Supplementary material for: Natural variation in SlSOS2 promoter hinders salt resistance during tomato domestication
Source: Hortic Res. 2022 Oct 26;10(1):uhac244. doi: 10.1093/hr/uhac244 (PMC9832868; doi:10.1093/hr/uhac244)
Supplement: Web_Material_uhac244 [file web_material_uhac244.zip › Supplemental Table 2-R1.docx]

**Supplemental Table 2. Primers used in this study**

| **Name** | **Sequences** |
| --- | --- |
| *SlSOS2*-sgRNA1-FP | TGATTGAGCTTGGGAAGTATGAAGT |
| *SlSOS2*-sgRNA1-RP | AAACACTTCATACTTCCCAAGCTCA |
| *SlSOS2*-sgRNA2-FP | TGATTGTTGCACGAAACACCGAGAC |
| *SlSOS2*-sgRNA2-RP | AAACGTCTCGGTGTTTCGTGCAACA |
| *SlSOS2pro*-FP | AAGCTTTTAAAATATTGAATTCATCACACAC |
| *SlSOS2pro*-RP | ACTAGTCACTGAACACTCATTTCCCACAG |
| ABI4-MBP-FP | ATGGACCCTTTAGCTTCCCAA |
| ABI4-MBP-RP | ATAGAATTCCCCCAAGATGGG |
| *SlSOS2pro*-EMSA-FP | CTCTAAAATTGTTTTGATTGTCGGTG |
| *SlSOS2pro*-EMSA-FP | AGGCTATTATCGATAAACCAATGATCG |
| *SlSOS2*-qFP | TTGCCTCAACAAGGAGTCGAGCT |
| *SlSOS2*-qRP | GCAGCCTTGATCTTTGTATACAAGGTAGG |
| *SlEF1α*-qFP | GACAGGCGTTCAGGTAAGGA |
| *SlEF1α*-qRP | GGGTATTCAGCAAAGGTCTC |

Note: FP, forward primer; RP, reverse primer; Restriction enzyme cleavage sites are underlined.
