# Supplementary figures and images for: Natural variation in SlSOS2 promoter hinders salt resistance during tomato domestication
Source: Hortic Res. 2022 Oct 26;10(1):uhac244. doi: 10.1093/hr/uhac244 (PMC9832868; doi:10.1093/hr/uhac244)

Supplemental Figure 1

A

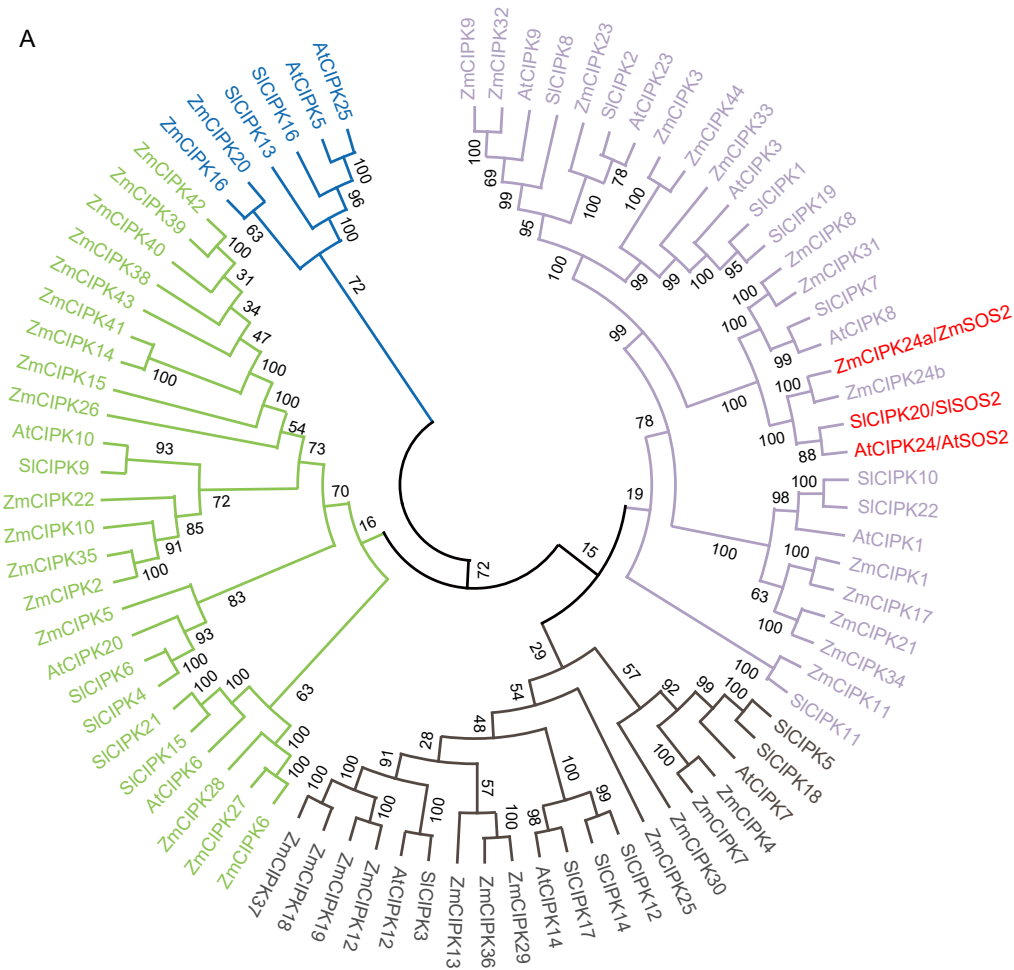

Supplement: Web_Material_uhac244 [file web_material_uhac244.zip › Supplemental Figure 1.pdf]

Supplemental Figure 2

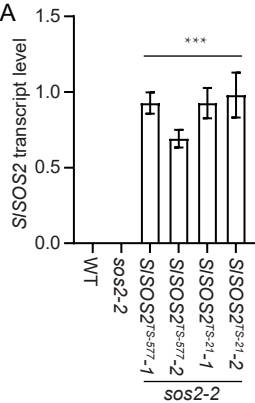

Supplement: Web_Material_uhac244 [file web_material_uhac244.zip › Supplemental Figure 2.pdf]
